# Nutrition Optimization Among Critically Ill Patients in the Cardiac Intensive Care Unit

**DOI:** 10.1007/s11886-025-02208-9

**Published:** 2025-04-04

**Authors:** Saisnigdha Allaparthi, Amanda Bode, Christan Bury, Amanda R. Vest

**Affiliations:** 1 Tufts University School of Medicine, Boston, MA USA; 2 Cleveland Clinic, Cleveland, OH USA; 3 Department of Cardiovascular Medicine, Heart, Vascular and Thoracic Institute, Cleveland Clinic, Cleveland, OH USA

**Keywords:** Malnutrition, Muscle Wasting, Cardiac Intensive Care Unit, Parenteral Nutrition, Enteral Nutrition, Testosterone Analogs

## Abstract

**Purpose of Review:**

Many critical care clinicians are unfamiliar with management principals or recent studies that guide nutritional optimization of patients in the cardiac intensive care unit (CICU). The goal of this review is to describe the prevalence of malnutrition in the CICU, the frameworks for malnutrition diagnosis and assessment of skeletal muscle wasting, and the potential clinical consequences of improper feeding practices.

**Recent findings:**

Malnutrition is common and has been linked to poor outcomes across various CICU patient populations. Several nutritional randomized controlled trials have refined best practices around the timing of enteral nutrition and the selection of protein intake targets in the intensive care setting. A hypocaloric, rather than normocaloric, feeding regimen usually preferred during the early phase of critical illness, and it is important to await adequate gut perfusion before uptitrating enteral feeds.

**Summary:**

There is an evolving evidence base that defines current practice in CICU nutritional management, albeit with multiple knowledge gaps warranting further study.

## Introduction

Patients in the Cardiac Intensive Care Unit (CICU) suffer from a range of cardiovascular conditions, influenced by various demographic factors and comorbidities. The primary diagnoses of patients in the CICU include acute coronary syndrome (ACS), acutely decompensated heart failure (HF), cardiogenic shock (CS), post-cardiac surgery, and unstable arrhythmias including cardiac arrest [1]. In addition, these patients tend to present with comorbidities including but not limited to hypertension, diabetes mellitus, acute kidney injury (AKI) in some cases requiring renal replacement therapy (RRT), and liver injury or cirrhosis [1]. The prognosis of patients in the CICU is strongly influenced by factors such as baseline comorbidity burden and the skeletal muscle wasting syndromes cachexia and sarcopenia [2, 3]. Critical illness is a period of extreme vulnerability for patients of all ages, and can exacerbate pre-existing malnutrition or provoke new nutritional deficiencies during the acute care course [4].

Nutritional optimization strategies for critically ill patients in the CICU may strongly influence the rate and extent of recovery during the hospitalization and beyond. The timing, type, and rate of nutritional support are recognized as modifiable factors that may affect the pace recovery of critically ill patients and may be more precisely facilitated by estimates of acute caloric requirements. This review discusses evidence-based approaches that may be employed to help support patients in the CICU with varying conditions and clinical courses.

## Understanding Caloric Requirements in Critically Ill Patients

During the early phase of critical illness, there is mobilization of caloric reserves in the body, with breakdown of glycogen, lipid stores and muscle proteins to produce glucose. During this acute metabolic response, amino acids exit the muscle to meet the need for more acute-phase proteins [5, 6]. However, it does not appear possible to prevent this energy mobilization by enhancing exogenous calorie delivery in the first few days of ICU admission and full nutritional support in the early phase of critical illness may be potentially harmful due to the risk of refeeding syndrome [7, 8]. Therefore, a hypocaloric feeding regimen usually chosen for the initial phase of critical illness rather than normocaloric feeding [9]. As illnesses progress, energy expenditure increases and exogenous nutrition becomes important, thus allowing for more nutritional support. As a result, current nutrition guidelines recommend a gradual uptitration in caloric intake over the first 3–5 days of CICU admission to avoid overfeeding [10]. From day 5 it is generally considered reasonable to target full energy expenditure requirements with exogenous feeding.

Refeeding syndrome describes the pathophysiological consequences of sudden resumption of feeding after a prolonged period of undernutrition, especially if carbohydrate-rich enteral or parenteral nutrition is provided. The acute rises in circulating glucose availability prompts the release of insulin, which drives electrolytes such as phosphorous and potassium into the cells. This can provoke severe hypophosphatemia and hypokalemia that can be life-threatening. CICU clinicians should recognize the potential for refeeding syndrome in patients who have been without adequate nutrition for several days or more, and reintroduce macronutrients cautiously with meticulous electrolyte monitoring and repletion [11].

Determining the caloric requirement in critically ill patients from day 5 onwards is a core component of CICU nutritional support. Predictive equations can be used to calculate caloric requirements (e.g. Harris-Benedict [12], Mifflin-St. Jeor [13], World Health Organization [14]). However, their reliability in acutely ill patients is uncertain and even within outpatient cardiac populations, for example in outpatients with heart failure with preserved ejection fraction (HFpEF), predictive equations have been shown to overestimate resting energy expenditure (REE) [15]. Indirect calorimetry (IC) is considered the gold standard for assessing energy requirements in critically ill patients by collecting inspired and expired gas, thus enabling analysis of oxygen consumption (VO_2_) and carbon dioxide (VCO_2_) production and calculation of the metabolic rate via the Weir Eq. [16, 17]. Metabolic carts can be connected to a disposable flowmeter that attaches to the endotracheal tube in a mechanically ventilated patient, which allows for the measurement of ventilator parameters along with VO_2_ and VCO_2_ measurements [18]. It has been demonstrated that IC performed using metabolic carts can accurately assess VO_2_ and VCO_2_ in mechanically ventilated patients, making them a more reliable approach to REE calculation than the use of predictive Eqs. [18]. A facemask or hood attachment is used for patients who are not mechanically ventilated. Limitations to IC include issues such as air leaks, high positive pressure ventilation, fraction of inspired oxygen (FiO_2_) over 80%, and other gases that can create unreliable results [17]. Furthermore, patients who require continuous renal replacement (CRRT) and extracorporeal membrane oxygenation (ECMO) may have inaccurate IC measurements due to non-respiratory carbon dioxide removal [17]. It may be necessary to repeat IC more than once during a prolonged CICU stay if ventilation or mechanical circulatory support status significantly changes over time.

### Diagnosis, Prevalence, and Implications of Malnutrition in the CICU

Malnutrition is defined as deficiencies, excesses, or imbalances in a person’s intake of energy and/or nutrients [19]. Undernutrition is characterized by deficiencies of micronutrient or macronutrient intake that result in abnormalities of physiology and body composition [20]. Malnutrition is associated with a high risk of morbidity and mortality in critically ill patients, with a worldwide prevalence of malnutrition in ICU patients being 38% to 78% [21]. The prevalence of malnutrition is pertinent across individuals with various cardiac conditions, but there are limited studies surveying malnutrition prevalence across multiple cardiac conditions [22]. A meta-analysis assessing patients diagnosed with ACS showed that of the 37,303 patients, 33.5% had malnutrition [23]. In addition, another meta-analysis of patients with HF showed that out of 12,537 subjects, 46% were malnourished [24].

Multiple medical record-based risk scores and questionnaire-based screening tools are available to screen a patient’s malnutrition risk. Medical recorded-based scores include the nutritional risk score (NRS) [25], geriatric nutritional risk index (GNRI) [26], prognostic nutritional index (PNI) [27], controlling nutritional status score (CONUT) [28] and the modified nutrition risk in critically ill (m-NUTRIC) [29] that was developed and validated specifically for the ICU population [30]. Tools requiring patient questioning or physical examination beyond that routinely available in the medical record include the malnutrition universal screening tool (MUST), malnutrition screening tool (MST), mini nutritional assessment (MNA), short nutritional assessment questionnaire (SNAQ), and subjective global assessment (SGA) [30]. A literature review of malnutrition assessment tools in older patients with aortic stenosis, coronary artery disease (CAD), and HF was conducted to discern which screening strategy was most effective in stratifying risk. The authors concluded that the Mini Nutritional Assessment-Short Form (MNA-SF) was easy to perform and had good predictive value for patients with aortic stenosis [31]. For patients with acute coronary syndromes (ACS), CONUT or MNA-SF were recommended [31]. Patients with HF in the outpatient or emergency department settings have shown good risk stratification from the PNI, GNRI (geriatric nutritional risk index), and MNA tools [31], whereas the GNRI had the best prognostic performance in a pre-heart transplant setting [32]. Assessing a patient’s malnutrition risk can provide insight into the patient’s overall prognosis and likelihood to benefit from nutritional intervention, and can help direct registered dietitian (RD) resources towards the patients with the greatest needs for a fuller nutritional assessment and potential intervention.

Malnutrition prevalence highly depends upon the criteria or guidelines used to define the diagnosis. The Academy of Nutrition and Dietetics and American Society of Parental and Enteral Nutrition (ASPEN) indicators for the diagnosis of adult and pediatric malnutrition (AAIM) is a method used by RDs to diagnose malnutrition by evaluating the following six characteristics: weight, intake, muscle, fat, functional status, and fluid level [33]. The Global Leadership Initiative on Malnutrition [34, 35] was also convened to establish guidelines for malnutrition diagnosis [34, 35]. The basic principles are similar to AAIM, as GLIM evaluates etiologic criteria including inflammation, reduced intakes, and criteria regarding weight loss and a physical exam. GLIM is not meant to replace the AAIM guidelines but rather to supplement them. Additionally, GLIM is an excellent tool for institutions where access to an RD is limited and the physicians or independent practitioners are evaluating for malnutrition without the help of a credentialed dietitian. The comparisons of AAIM and GLIM can be seen below and additional validation is ongoing [36]:

The GLIM criteria indicate that a diagnosis of malnutrition can only be made if at least 1 phenotypic and etiologic criterion are met [34, 35]. Furthermore, the severity grading of malnutrition can be divided into stage 1 and stage 2 malnutrition based on the phenotypic criteria of unintentional weight loss, low BMI, and reduced muscle mass. Stage I (moderate) malnutrition is characterized by 5–10% weight loss within the past 6 months or 10–20% beyond 6 months [34, 35]. Stage 2 (severe malnutrition) is characterized by greater than 20% of body weight loss beyond 6 months [34, 35]. In contrast, the AAIM criteria define moderate/severe malnutrition specific to the following three scenarios: acute care setting, chronic disease, and social circumstances [33, 37]. Each scenario has corresponding cutoffs based on the AAIM criteria listed in Table 1 [37].

**Table 1:** Global Leadership Initiative on Malnutrition and Academy and ASPEN Indicators of Malnutrition Criteria

Criteria	GLIM [34, 35]	ASPEN/AAIM [33]
Low BMI*	BMI < 18.5 kg/m^2^ (cut-offs may vary by age and health status)	Not applicable
Reduced Muscle Mass*	Validated imaging techniques preferred. Physical assessment allowable as surrogate	Physical Assessment: Muscle wasting of the temples, clavicles, shoulders, interosseous muscles, scapula, thigh and calf
Unintentional Weight Loss*	Weight loss > 5% in the last 6 months or > 10% in the past year	Weight loss parameters specific to etiology. evaluated considering other clinical findings, including hydration status
Reduced Food Intake or Absorption^	Includes inadequate calorie intake, gastrointestinal absorption issues, or conditions leading to decreased appetite	Energy intake evaluated based on diet history, calculating energy and protein demand
Inflammatory Response^	Evidence of inflammation (e.g., elevated C-reactive protein levels or other systemic markers)	Inflammatory response drives etiology of malnutrition (i.e. Acute, Chronic or Social/Environmental)
Reduced Body Fat	N/A	Loss of subcutaneous fat assessed by physical assessment
Fluid Accumulation- Physical Assessment	N/A	Weight loss is marked by generalized fluid retention (edema) and fluid weight gain may be observed
Functional Assessment	N/A	Grip Strength measured

*: GLIM Phenotypic Criteria

^: Etiologic Criteria

### Muscle Wasting Syndromes in Cardiac Conditions

Muscle wasting in HF is thought to be driven by a catabolic state that leads to muscle protein degradation, systemic inflammation activation, and reduced caloric intake due to anorexia [3]. While REE is generally increased in patients with cancer cachexia, it is unclear whether REE is increased in cardiac cachexia. Indirect calorimetry is calculated using VO_2_ as a proxy for tissue energy needs. However, because the VO_2_ is often low in decompensated HF due to low cardiac output, it is possible that the REE may calculate as lower than expected due to poor tissue delivery of blood flow [38, 39]. Regardless of whether or not the REE is elevated in CICU patients with muscle wasting, the end result is a shift in the balance between muscle protein synthesis that ultimately favors muscle degradation [38, 39]. The relative contributions of a cachectic metabolic state and inadequate dietary macronutrient intake are uncertain [40].

Cachexia and sarcopenia are two muscle-wasting conditions that negatively affect outcomes in HF. Sarcopenia is defined as the age-related decline in skeletal muscle mass or quality, leading to reduced exercise tolerance and increased morbidity, which can be accelerated in the setting of medical conditions [41]. Sarcopenia, as defined by lean mass assessments using dual X-ray absorptiometry (DXA), has been associated with increased mortality in ambulatory HF populations [40]. Cardiac cachexia is a catabolic condition that develops in chronically or acutely in patients with HF, characterized by unintentional weight loss, decreased appetite, and inflammation [40]. Cachexia is independently associated with mortality in patients with HF. According to the seminal European report from 1993 to 1995, 16% of 171 outpatients with HF had cachexia based on a ≥ 7.5% unintentional weight loss over at least 6 months. The subgroup with cachexia had a 50% mortality rate at 18 months, versus 17% for those without cachexia [40].

### Skeletal Muscle Ultrasound

The measurement of lean body mass is an important metric of nutritional assessment in a patient [42]. Skeletal muscle ultrasound (SMUS) is a technique that has been used at the bedside to help discern nutritional risk In a systematic review of 37 studies, incorporating 3100 patients, of which 22 were conducted in critical care settings, 76% of studies found that SMUS metrics including cross-sectional area, muscle thickness, and echointensity, showed significant associations with functional capacity, length of stay, readmission, and survival assessment [42]. Bedside US may be more sensitive to muscle changes in the short term, especially in populations with BMI > 35 kg/m^2^ where muscle changes can be masked by subcutaneous fat and fluid accumulation. Objective guidelines outlining technique and interpreting changes have yet to be established, though if the consistency of variables are controlled, bedside US appears to be an accurate way to assess muscle changes [43–45]. Increasingly, critical care RDs are utilizing SMUS to complement the standard malnutrition diagnostic criteria to obtain a fuller impression of body composition and nutritional status.

### Skeletal Muscle Computed Tomography

The evaluation of skeletal muscle mass can also be opportunistically evaluated in patients with abdominal or chest computed tomography (CT) scans. Single slice skeletal muscle area (SMA) at the third lumbar vertebra (L3) is used to correlate to whole body muscle mass [46], however patients in the CICU are more likely to have a chest CT that is opportunistically available. When comparing anatomical areas in chest CT compared to abdominal CT, the twelfth lumbar vertebra (T12) had the strongest correlation to L3 (r = 0.834, *p* < 0.001) when compared to other anatomical areas [47]. The skeletal muscles at T12, the rectus abdominis, diaphragm, external oblique, intercostals, latissimus dorsi and erector spinae are segmented to find the area and normalized to height to compare to normative data [48]. Pectoralis muscle mass and quality per CT assessment has demonstrated prognostic significance in LVAD cohorts [49, 50]. Limitations of utilizing CT scans for diagnosing sarcopenia and malnutrition are the availability of abdominal or chest CT scans, the potential impact of intravenous contrast use, and the imaging skillset required to acquire regional muscle mass measures from CT images.

### Selecting Nutritional Plans for Patients in the CICU—Parenteral vs Enteral Nutrition

Selecting the correct course of nutritional intervention is vital to rehabilitating patients in the CICU. Enteral nutrition is preferred for patients as it is known to have a positive impact on the intestinal mucosa and fewer side effects associated with it, unlike parenteral nutrition [51]. Parenteral nutrition is associated with greater potential for bloodstream infection, electrolyte disturbances, overfeeding, and vascular complications [51]. It is typically reserved for patients suffering from gastrointestinal motility issues such as ileus or those with a prolonged period of nil per os (NPO) status. A principle adopted from the general ICU setting to the CICU is the benefit of early institution of feeding, meaning within 48 h of hospital admission [52]. If enteral nutrition cannot be started within 7 days of admission, or if < 70% of caloric intake can be delivered through the enteral route, parenteral nutrition (PN) is usually indicated. PN is sometimes started before day 7 in severely malnourished patients [11, 52]. Key differences in indications, timing, complications, and monitoring required for parenteral and enteral nutrition are outlined in Table 2.

**Table 2:** Enteral versus Parenteral Nutrition Guidance

	Enteral Nutrition	Parenteral Nutrition
Preferred Use	- First-line choice when the GI tract is functional, and patient is hemodynamically stable [51] - Patients can be administered enteral nutrition on 0.14 and 0.3 µg/kg/day norepinephrine or its equivalent of vasopressors [53]	- Used when GI tract is non-functional [51] (ileus, delayed gastric emptying, hyperemesis, excessive ostomy output, recurrent small bowel obstruction)
Indications	- Patient is at risk of malnutrition	- Severe GI issues - Failure of EN after 5–7 days
Timing	- Start within 24–48 h if feasible [51]	- Start TPN after 7 days in well-nourished patients [54] - Start TPN in 3–6 days in at risk malnutrition patients [54]
Complications	- Aspiration of tube feeds leading to aspiration pneumonia - Worsening mesenteric ischemia in the setting of an increased need for oxygen for enteral feeding - Risk of refeeding syndrome	- Increased risk of infections, fluid, central line infections, refeeding syndrome, hyperglycemia [51]
Monitoring	- Monitoring of electrolytes and fluid balance - Signs of GI intolerance (constipation, nausea, and vomiting)	- Requires more strict monitoring of patient’s electrolytes and fluid balance - Need to monitor for signs of infection

Patients requiring vasopressors warrant special consideration when determining their nutritional regimen. Vasopressors can induce splanchnic hypoperfusion, which puts patients at risk for gut ischemia if enteral nutrition is started prematurely [51]. However, data suggests that enteral nutrition can still be administered safely to patients receiving lower-dose vasopressors between 0.14 and 0.3 µg/kg/day norepinephrine or its equivalent [53]. Furthermore, patients on vasopressors should be started with the slow advancement of trophic feeds. Trophic feeding (10–20 ml/hour with hypocaloric energy provision) should be maintained with additional PN until the resumption of gut function is reached.

### Protein Intake Targets in the CICU

Nutritional interventions remain a topic of study for patients in the ICU, especially those receiving mechanical ventilation. In the PRECISe trials, mechanically ventilated patients were provided with either higher enteral protein intake (2.0 g/kg per day) or standard provision (1.3 g/kg per day) to evaluate for potential improvements in quality of life with the higher protein intake [55]. Of the 935 patients, approximately a quarter were admitted to the ICU for cardiovascular indications. The primary endpoint was the EQ-5D-5L health utility score assessed at 30 days, 90 days, and 180 days after randomization, which was found to be lower (less favorable health status) for the high protein group than the standard protein group. Higher enteral protein intake was also associated with more gastrointestinal intolerance [55]. Another clinical trial called Effect of Higher Protein Dosing in Critically Ill Patients With High Nutritional Risk (EFFORT Protein) studied ≥ 2.2 g/kg/day enteral protein versus ≤ 1.2 g/kg/day administration in high-risk mechanically ventilated patients. The trial showed no statistical significance between both groups in relation to the primary outcome (time to discharge alive) and secondary outcome (60-day mortality rate). Furthermore, this study suggested higher mortality with greater protein provision among patients with renal failure [56]. Another protein target study incorporated a large proportion of patients with HF and cardiac indications for ICU admission. The participants were randomized to receive either 20 kcal/kg/day and protein 1.8 g/kg/day or 20 kcal/kg/day and protein 0.9 g/kg/day regimens, with both nutritional protocols becoming the same at day 10. There was lesser femoral muscle volume loss with the higher-protein intervention during electrical muscle stimulation [57].

### Nutritional Support for Patients with Acute Heart Failure

Patients with acute HF comprise a substantial portion of CICU populations. Addressing malnutrition in the HF population has been shown to decrease 30-day mortality in hospitalized patients with chronic HF in a substudy of the open-label Effect of Early Nutritional Therapy on Frailty, Functional Outcomes and Recovery of Undernourished Medical Inpatients Trial (EFFORT) study [58]. Current consensus suggests that a protein intake goal of 1.1 g/kg/day may benefit patients at risk of cardiac cachexia while also considering the risk of renal disease progression and the effects of animal protein sources on CAD progression [40]. However, no robust prospective data supports an optimal protein intake range for patients with chronic HF, nor those hospitalized with acutely decompensated HF. There is also insufficient evidence to support specific nutritional supplements or medications to promote muscle mass maintenance or recovery in patients with heart failure with reduced ejection fraction (HFrEF) [40]. The most dramatic examples of cachexia reversal are seen after treatment of advanced HFrEF with heart transplantation or left ventricular assist device (LVAD) implantation [40]. The months after LVAD implantation have been associated with reductions in insulin resistance and inflammation, and increase in cholesterol and albumin levels [40]. LVAD recipients tend to gain weight during device support, especially if the pre-LVAD body mass index (BMI) is under 25 kg/m^2^ [2, 40]. According to a single center LVAD cohort, HFrEF improvement characterized by a reduction in N terminal pro B type natriuretic peptide (NT-proBNP) levels pre-dated an increase in BMI, and is hypothesized to be necessary for muscle mass recovery [40]. There is a lack of efficacy evidence and objective metrics to track the effect of nutritional interventions for the potential treatment of cardiac cachexia [40].

Malnutrition in the setting of a recent hospitalization for HF was also investigated in the PICNIC trial, with the patients receiving an individualized nutritional intervention in addition to conventional HF treatment showing lower readmission and mortality rates [51]. Nutritional support for patients with HF in the CICU is generalized to include practices such as early nutritional assessment, determining the form of administration, timing, and dose of nutrition, and assessment of nutritional support [51]. This can include limiting salt and fluid intake at times based on the presence of fluid congestion, and potentially supplementation with trace elements and multivitamins, including intravenous iron for those with iron deficiency and thiamine (vitamin B1) in patients receiving high-doses of loop diuretics [51]. Amongst patients with HF, enteral nutrition is again favored due to its positive impact on the intestinal mucosa and lower side effect profile [51].

### Nutritional Support for Patients with Acute Coronary Syndrome (ACS)

Emerging evidence suggests that malnutrition is associated with a poor prognosis among patients with ACS [23]. A meta-analysis of 30 studies comprising over 37,000 patients with ACS in which 33.5% had malnutrition showed that malnutrition was significantly associated with all-cause mortality risk following ACS, regardless of ACS type, ethnicity, and income status: malnourished patients with ACS had a 2.6-fold higher risk of mortality [23]. It was also found that malnourished patients who received nutritional intervention after admission for AMI showed similar all-cause mortality to the well-nourished patients.

However, no specific guidelines are in place for malnutrition management in patients recovering from Acute Coronary Syndrome (ACS), especially in the acute phase post-MI. Dietary patterns such as the DASH (Dietary Approaches to Stop Hypertension), Mediterranean style diet are broadly recommended by the American Heart Association (AHA) for secondary prevention of cardiovascular events [59]. Guidelines focus on adjusting energy intake and expenditure to achieve and maintain and healthy body weight, with the avoidance of processed foods and replacement of saturated fats with polyunsaturated and monounsaturated fats. AHA guidelines for the primary prevention of atherosclerotic cardiovascular disease also endorse minimizing the intake of trans fats, red meat and processed red meats, refined carbohydrates, and sweetened beverages. However, striking a balance between adequate dietary intake and avoidance of foods that may promote CAD can be challenging for malnourished patients, who require adequate caloric intake and protein intake to regain physical strength and optimize prognosis. Therefore, further evidence is needed to help discern what the best intervention is for malnourished patients recovering from ACS.

### Nutritional Support in Cardiogenic Shock

The pathophysiology of low cardiac output in the setting of cardiogenic shock could lead to intestinal hypoperfusion, making this patient population susceptible to mesenteric ischemia [51]. This can be further exacerbated by vasopressors as vasopressors can further direct blood away from mesenteric vasculature [51]. Furthermore, mesenteric ischemia can be worsened by the increased demand for oxygen needed to perform absorption and digestion when a patient is provided enteral feeding [51]. This concept is supported by the NUTRIREA-2 and NUTRIREA-3 randomized control trials, which tested early full-dose nutrition (20–25 kcal/kg/day) within 24 h of intubation in ventilated patients with shock (mean dose of norepinephrine of 0.53 µg/kg/day, administered to 1 in 5 patients with cardiogenic shock) against controls [51]. In these RCTs, the enterally fed intervention groups suffered from more bowel ischemia and feeding intolerance when compared to the control. However, in feeding protocols that are carefully monitored, where nutrition usually begins after 2–3 days and involves hypocaloric approaches in the early stages, the occurrence of confirmed bowel ischemia was very low, at less than 1% [51]. These protocols also included close monitoring for signs of mesenteric ischemia, leading to the interruption of enteral nutrition in about one-third of patients. Furthermore, recent results from NUTRIREA-3 suggest that starting at a low-calorie, low-protein feeding regimen compared to standard intakes (0.2–0.4 g/kg per day of protein vs 1.0–1.3 g/kg per day of protein) in the first days of acute critical illness has beneficial effects on cardiogenic shock patients [51].

Data regarding nutritional requirements for patients receiving temporary mechanical circulatory support (MCS) remains limited, but some specific observations have been reported. In a cohort of 65 patients receiving extracorporeal membrane oxygenation (ECMO) and EN, 36 (55.4%) received early EN [60]. Thirteen (36.1%) patients had EN intolerance in the early EN group, which was significantly lower than that in the delayed EN group (82.8%, *p* < 0.001). Nineteen patients (52.8%) survived in the early EN group, which was significantly higher than that in the delayed EN group (20.7%, *p* = 0.008), although confounding may have been present [60]. The authors concluded that EN started with hypocaloric doses within 48 h of ECMO initiation is reasonable. Another trial retrospectively assessed the optimal timing of feeding amongst critically ill patients with resuscitated cardiac arrest [61]. It was found that in patients with refractory out-of-hospital cardiac arrest (OHCA) treated with extracorporeal cardiopulmonary resuscitation (ECPR) and targeted temperature management (TTM), delayed enteral nutrition (> 48 h) was associated with increased neurologically favorable outcomes compared to the early enterally fed (< 48 h) group [61]. However, this was also a non-randomized cohort study and confounding may have influenced this finding.

Other temporary MCS platforms including the Impella, intra-aortic balloon pump (IABP), and Tandem Heart, also require cautious management of enteral nutrition. In a prospective study of critically ill patients post-cardiac surgery with extracorporeal circulation, 18 patients required an IABP, 58 patients required epinephrine, and 40 patients required artificial nutrition [62]. In this study, they found that enteral nutrient delivery was negatively affected by increasing dopamine and norepinephrine levels required by patients [62]. However, enteral nutrition was not affected by IABP. Another retrospective study from Japan analyzed the nutritional support needed during temporary MCS amongst 47 patients with a 30-day mortality rate of 32%. EN was delivered once hemodynamic stability was achieved with an energy goal for the first week of 25 kcal/kg of ideal body weight per day. There were EN complications in 24 (51%) of the MCS group, but no association between EN initiation during MCS and the 30-day mortality rate or EN complications [63].

### Nutritional Support for Cardiac Surgery Patients

Pre-existing malnutrition in cardiac surgery patients can be further exacerbated by preoperative fasting and the post-operative delay of nutritional support [64]. Furthermore, preoperative fasting has been shown to aggravate dyspnea, gut edema, and hepatic congestion [64]. While nutritional adequacy was low in cardiac surgery patients, it was not associated with reduced overall mortality. When comparing the effects of no nutrition, EN, or PN from 2 days before to 2 days after a coronary artery bypass graft (CABG) on myocardial atrial tissue samples, it was found that there were no significant differences in the myocardial inflammatory response between the different interventions [65].

In contrast, the caloric control in cardiac surgery patients (CoCoS) trial evaluated the influence of nutrition therapy on the outcomes of cardiac surgery patients. It was found that male patients in the NT (nutrition therapy) group had significantly less arrhythmia than in the retrospective CT (control therapy) group [66]. In contrast, females in the NT group had significantly less pneumonia than in the CT group [66]. Survival was significantly higher in female NT patients compared to CT patients, both for CABG and aortic valve surgery [66].

### Nutritional Approaches for Patients Pre Cardiac-Procedures

It is standard protocol for patients to remain NPO for 6–12 h before cardiac procedures. The cardiac Enhanced Recovery After Surgery (ERAS) guidelines endorse, where possible, supplementation with 7–10 days of intensive nutrition therapy pre-operatively for patients with a pre-cardiac surgery serum albumin level below 3.0 g/dL [67]. Consumption of clear liquids until 2 to 4 h preoperatively with a carbohydrate load of a 12-oz clear beverage or a 24-g complex carbohydrate beverage at 2 h preoperatively is an important component of all ERAS protocols outside cardiac surgery. However, due to the limited evidence in cardiac surgery populations these practices receive weaker class IIb recommendations for pre-cardiac surgery patients, per consensus level of evidence [67].

The SCOFF trial specifically assessed the need for fasting before cardiac catheterization procedures requiring conscious sedation [68]. The primary composite outcome of hypotension, aspiration pneumonia, hyperglycemia or hypoglycemia occurred in 19.1% in the fasting group and 12.0% in the no-fasting group, confirming the non-inferiority of no fasting [68]. In terms of secondary outcome events (contrast-induced nephropathy, new ICU admissions post procedure new ventilation requirements, 30-day mortality, 30-day readmissions) the SCOFF trial showed no apparent differences [68]. Patient satisfaction was better in the no-fasting group. The results of this trial, in conjunction with other similar small studies [69, 70], support reconsideration of NPO guidelines before coronary catheterization and device implantation procedures.

### Nutritional Requirements for Patients with CKD and ESRD

Patients with chronic kidney disease (CKD) have nutritional requirements specific to the protein quantity allowed in their diet. Current practice favors that adults with mild CKD (stages 1 and 2) should avoid high protein intake (> 1.30 g/kg/day) and patients with CKD stages 3–5 not on dialysis are recommended to restrict protein intake to 0.60–0.80 g/kg/day [71]. These recommendations stem from the Modification of Diet in Renal Disease (MDRD) study in which a dietary protein target of 0.6 g/kg/day appeared to limit progression of CKD across two cohorts of patients mostly without diabetes, at least during medium-term follow-up (6 years) [72, 73]. However, in a more recent observational study there were neutral or inverse associations between protein intake and mortality among patients with CKD stages 3–5 not on dialysis [74]. This retrospective analysis also suggested that higher protein intake was associated with a lower risk of all-cause death over 4 years despite participants being told to limit protein intake, indicating that the benefits of higher dietary protein intake may surpass the consequences of any disease acceleration [74].

Elevated protein catabolism and malnutrition are commonly observed for patients with end stage renal disease (ESRD), especially in the setting of suboptimal energy supply as ESRD [75]. This leads to patients with ESRD requiring a higher amount of energy intake than healthy individuals. REE is shown to increase from 12 to 20% during dialysis [75]. In non-dialysis CKD patients, a neutral or slightly positive nitrogen balance can be maintained with a low quantity (~ 0.6 g/kg/day), high-quality protein diet combined with adequate energy intake (30–35 kcal/kg/day) [75]. In patients on maintenance hemodialysis or peritoneal dialysis, the protein requirement is higher (1.2–1.3 g/kg/day) [75]. The higher protein requirement is due to dialysis-related protein loss, extra-energy expenditure, and persistent inflammation.

### Nutritional Requirement for Patients with Liver Cirrhosis

The general diet for a patient with cirrhosis should be high in protein and high in energy due to the catabolic effects of cirrhosis leading to increased protein degradation and muscle mass loss [76]. Furthermore, sodium intake should be restricted in patients with advanced disease, especially in the setting of ascites [76]. Muscle mass loss and to a greater extent sarcopenia have been associated with a worsening prognosis in cirrhosis patients [76]. It was shown that the loss of muscle mass in cirrhosis patients is associated with the development of hepatic encephalopathy when compared to a population of patients with cirrhosis who do not have sarcopenia [76]. Various nutritional therapies for cirrhosis, including parenteral nutrition (PN), enteral nutrition (EN), EN + intestinal probiotics, and late evening snacks (LES), have been studied. PN, EN, and PN + EN are routine therapies, while EN + probiotics and LES are emerging treatments [76]. Introducing a carbohydrate-rich snack (50 g of complex carbohydrates) shortens fasting time, eliminates the adverse changes resulting from night-time catabolism, and improves nitrogen metabolism, which leads to an increase in lean body mass and reverses sarcopenia [76]. According to the European Society for Clinical Nutrition and Metabolism (ESPEN), parenteral nutrition is recommended for moderate to severe cirrhosis patients who cannot eat or receive enough enteral nutrition, particularly if fasting exceeds 72 hours [76].

### Testosterone Analogs and ICU-Acquired Muscle Weakness

Testosterone analogs can activate androgen receptors in muscle tissue, leading to enhanced muscle growth, increased strength, and reduced ICU-associated muscle wasting (ICUAW) [77]. Testosterone effects on skeletal muscle mass occur via the mTOR pathway and androgen receptors to promote protein synthesis [77]. As a result, hypotestosteronemia may contribute to a net catabolic state, resulting in ICU-acquired weakness. Selective androgen receptor modulators (SARMs) can exert androgenic effects on specific tissues, which is advantageous in limiting the adverse effects of testosterone [78]. SARMs have been shown to increase lean body mass and bone density, thus making them hold promise for their potential to increase muscle mass and strength [78]. It was noted that certain SARMs such as GSK2881078 were well tolerated by healthy older males and post-menopausal females, with a greater dose-dependent response (increase in lean muscle mass) noted with a dose increase from 0.35 mg to 1.5 mg when compared to men [79]. Furthermore, women in this study did not suffer from clinical signs of virilization, suggestive of SARMs’ lower risk side effect profile as they cannot be aromatized into estrogen [79, 80]. Limited research is available comparing the effects of SARMs between premenopausal and post-menopausal women. SARMs have also been noted to help in the prevention and treatment of muscle wasting associated with cancer [81]. In a randomized, double-blind, placebo-controlled phase 2 trial, patients with NSCLC and colorectal cancer were randomly assigned to receive a placebo, enobosarm 1 mg or enobosarm 3mg [81]. It was found that both enobosarm at 1 mg and 3 mg were safe and well-tolerated in cancer patients when compared to the assigned placebo [81]. Enobosarm also demonstrated a dose-dependent increase in lean body mass when given to 120 healthy elderly individuals [41]. It is associated with a decrease in insulin resistance and a favorable safety profile [41]. While no adverse effects have been noted with enobosarm, further phase III studies are needed to evaluate its safety and efficacy.

Alternatively, the oral androgen and synthetic anabolic steroid oxandrolone was initially approved for treating catabolic conditions caused by long-term corticosteroid therapy, severe burns or to treat bone pain associated with osteoporosis. There was some off-label use in ICUs beyond the burns indication to promote muscle mass regain in patients experiencing severe cachexia. Side effects associated with its use include peliosis hepatis, liver failure, intra-abdominal hemorrhage, liver cell tumors, increased risk of atherosclerosis, and increased prostatic hypertrophy and carcinoma in elderly patients [82]. Oxandrolone was used to treat 1-year post-burn pediatric patients with success [83]. It was found that oxandrolone improved the long-term recovery of severely burned children in height, bone mineral composition, cardiac work, and muscle strength [83]. It was found that oxandrolone reduced resting energy expenditure, increased IGF-1 secretion during the first year after burn injury, and led to an overall increase in lean body mass and muscle strength when combined with exercise [83]. Oxandrolone was initially considered to have a more acceptable adverse event profile than other anabolic steroids with less virilizing effects and lower risks of liver and cholesterol abnormalities. However, the company Gemini voluntarily requested to the FDA in 2023 that oxandrolone 2.5 mg and 10 mg be withdrawn from the market due to concerns about safety or efficacy [82].

Ghrelin agonists have also been trialed for their capacity to increase lean mass in the setting of medical illness. Anamorelin 50–100 mg for 12 weeks showed a mean increase of 1.1 kg of fat free mass versus placebo among patients with lung cancer cachexia [84], but this medication did not receive regulatory approval due to a lack of significant functional improvements. A promising pharmacological approach to cachexia recovery is inhibition of growth differentiation factor (GDF)−15, for example with the monoclonal antibody ponsegromab. Amongst 187 patients with non-small-cell lung, pancreatic, or colorectal cancer receiving ponsgromab 100–400 mg vs placebo administered subcutaneously every 4 weeks for three doses, subjects in the ponsegromab groups had significantly greater weight gain than those in the placebo group, with a median between-group difference of 2.8 kg in the 400-mg group [85]. Improvements in appetite, cachexia symptoms and physical activity were also observed in the 400 mg ponsegromab group, as compared to placebo. Further studies in cardiac populations are awaited.

## Conclusions

**Fig. 1:**
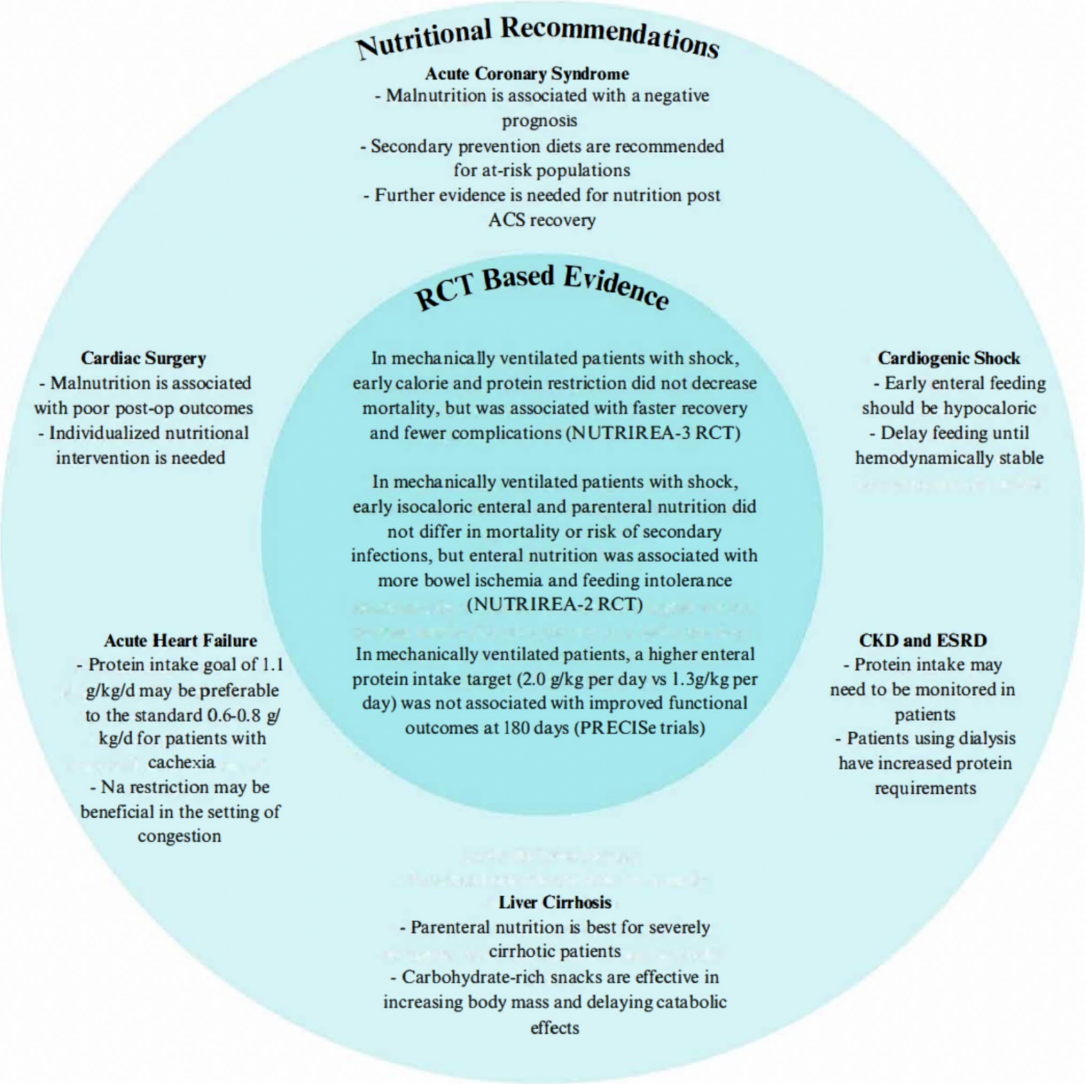
RCT-based and condition-specific recommendations [Key: ACS: acute coronary syndrome; CKD: chronic kidney disease; ESRD: end-stage renal disease; RCT: Randomized controlled trials]

Personalized nutritional intervention is vital to patient recovery and survivorship in the cardiac ICU. Malnutrition exacerbates the risk of multiple cardiac-associated conditions, but nutritional support requires a nuanced approach that is grounded in recent clinical trial results. Figure 1 summarizes foundational RCT-based knowledge and highlights specific nutritional approaches to consider certain for CICU conditions. Depending on the severity of a patient’s condition, differing methods can be used to calculate a patient’s metabolic requirements ranging from predictive equations to indirect calorimetry and the use of skeletal muscle imaging techniques. Fine-tuning the balance between delivering adequate nutritional support while avoiding the potential adverse consequences of enteral or parenteral nutrition is essential for optimizing the prognosis of patients admitted to the CICU.

## Key References

- Pascal Frederiks MP, Alexander Wilmer. Nutritional support in the Cardiac Intensive Care Unit. *European Heart Journal*. 2023;13.
  - This study highlights the critical role of malnutrition in shaping cardiac patients’ prognoses, emphasizing the need for evaluating nutritional status before initiating interventions,
- Onyedika J. Ilonze LPR-B, Rebecca Cogswell, Amy Hackman, Khadijah Breathett, Edward Saltzman, Amanda R. Vest. Controversies and Conundrums in Cardiac Cachexia. *JACC: Heart Failure*. 2024.
  - This study highlights the impact of cardiac cachexia on heart failure patient mortality, underscoring the need for improved strategies in its identification and management.

## Data Availability

No datasets were generated or analysed during the current study.
